# Reduced anxiety is associated with the accumulation of six serotonin reuptake inhibitors in wastewater treatment effluent exposed goldfish *Carassius auratus*

**DOI:** 10.1038/s41598-017-15989-z

**Published:** 2017-12-05

**Authors:** D. B. D. Simmons, E. S. McCallum, S. Balshine, B. Chandramouli, J. Cosgrove, J. P. Sherry

**Affiliations:** 1 Aquatic Contaminants Research Division, Water Science and Technology Directorate, Environment Canada, Burlington, ON Canada; 2 Department of Psychology, Neuroscience & Behaviour, McMaster University, Hamilton, ON Canada; 3 Metabolomics Services, SGS AXYS, Sidney, BC Canada

## Abstract

Pharmaceuticals and personal care products (PPCPs) have been found in wastewater treatment plant (WWTP) effluents and their recipient watersheds. To assess the potential of WWTP effluents to alter fish behaviour, we caged male goldfish (*Carassius auratus*) for 21-days at three sites along a contamination gradient downstream from a WWTP which discharges into Cootes Paradise Marsh, on the western tip of Lake Ontario. We also included a fourth caging site as an external reference site within Lake Ontario at the Jordan Harbour Conservation Area. We then measured concentrations of PPCPs and monoamine neurotransmitters in caged goldfish plasma, and conducted behavioural assays measuring activity, startle response, and feeding. We detected fifteen different PPCPs in goldfish plasma including six serotonin reuptake inhibitors (amitriptyline, citalopram, fluoxetine/norfluoxetine, sertraline, venlafaxine, and diphenhydramine). Plasma concentrations of serotonin were significantly greater in plasma of fish caged closer to the WWTP effluent outfall site. The fish caged near and downstream of the WWTP effluent were bolder, more exploratory, and more active overall than fish caged at the reference site. Taken together, our results suggest that fish downstream of WWTPs are accumulating PPCPs at levels sufficient to alter neurotransmitter concentrations and to also impair ecologically-relevant behaviours.

## Introduction

Wastewater treatment plant (WWTP) effluent is a ubiquitous source of anthropogenic contamination in aquatic ecosystems^1^. While there is a growing body of literature demonstrating that WWTP effluents can affect fish physiology and reproduction in the wild, the potential for WWTP effluent to affect ecologically important behaviours of aquatic organisms has not been widely addressed. WWTP effluents may impact behaviour because they contain a complex mixture of contaminants, many of which are endocrine and neurologically active^2,3^. In particular, pharmaceuticals and personal care products (PPCPs) that are present in WWTP effluents have emerged as one group of contaminants with the potential to alter animal behaviour. Psychotropic drugs are specifically designed to modulate human neurophysiology, which often affects behaviour. Because the biological targets of these drugs are conserved in teleost fishes, there is growing concern that chronic exposures to the PPCPs regularly found in WWTP effluents might affect fish and other aquatic vertebrates^4^.

The use of behaviour to assess the effects of chronic exposure to environmental contaminants in fish is gaining momentum in aquatic toxicology^5,6^. Behavioural responses could offer a more sensitive measure of exposure-driven effects compared to traditional endpoints because behaviour can be modified long before survival is impacted or physiological dysfunction is detected^7^. Additionally, changes in behaviour can be observed in a relatively non-invasive manner. Contaminant induced behavioural impairment can also have serious ecological implications - affecting reproduction, foraging, predation risk, and survival^8,9^. To date, a small number of studies have shown that exposure to WWTP effluent can indeed alter fish behaviour under controlled laboratory conditions. For example, Garcia-Reyero *et al*.^10^ and Martinović *et al*.^11^ showed that male fathead minnows (*Pimephales promelas*) were less able to compete for and hold a nesting site against unexposed rival males after a three-week laboratory exposure to 100% WWTP effluent. Similarly, male three-spine stickleback (*Gasterosteus aculeatus*) exposed to 50% or 100% effluent for three weeks built fewer nests and had reduced courtship behaviours^12^. A 10-week exposure to WWTP effluent in the laboratory, however, had little effect on goldfish (*Carassius auratus*) spawning behaviours^13^. In contrast, one of the few studies conducted on fish in the wild demonstrated that male mosquitofish (*Gambusia affinis*) collected downstream from a WWTP outfall actually courted females more than male fish collected from a pristine site^14^. Since the known targets of many PPCPs present in WWTP effluents go beyond reproductive targets, it follows that effluent could also impact non-reproductive fitness-important behaviours (e.g. foraging, avoiding predators). For instance, Eastern mosquitofish (*Heterandria formosa*) had reduced swimming performance and altered diurnal swimming activity after a 96-hr exposure to WWTP effluents^15^, and round goby (*Neogobius melanostomus*) were less aggressive after 28 d exposure to WWTP effluent^16^.

**Figure 1:**
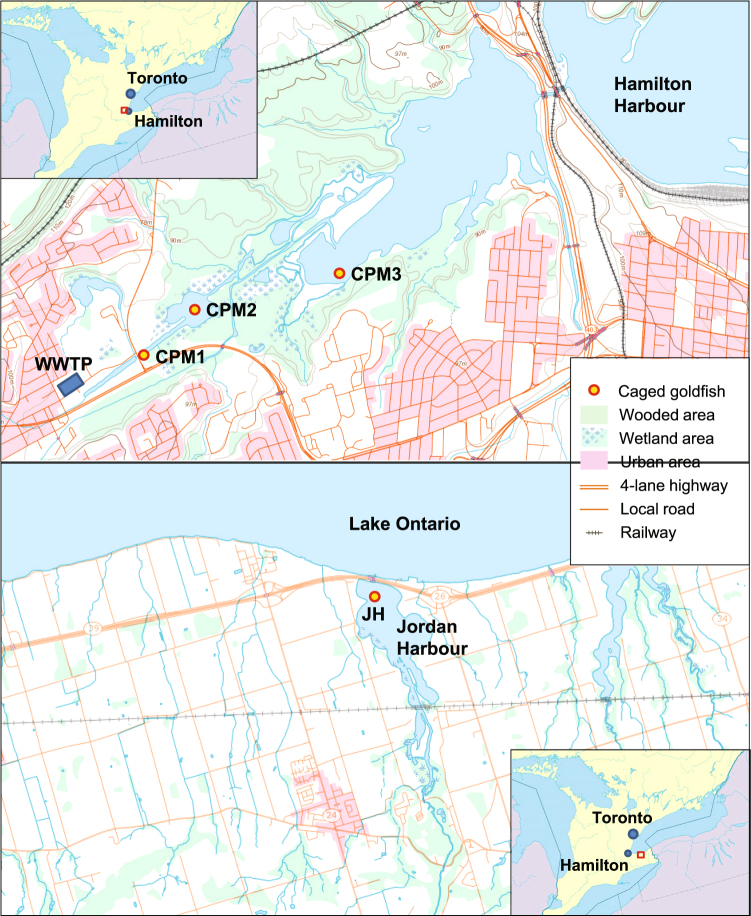
Caging locations in Cootes Paradise Marsh (CPM1, CPM2, and CPM3) and at Jordan Harbour (JH), Ontario, Canada. The base map is from the Atlas of Canada (with permission of Natural Resources Canada, http://open.canada.ca/en/open-government-licence-canada).

Thus, as the final part of our larger investigation to understand the effects of WWTP effluent and PPCPs on wild fish^17,18^, the present study’s aim was to assess how WWTP effluents affect fish behaviour. While laboratory exposures do not fully integrate ambient environmental conditions that fish experience in the wild, studies on field-collected animals cannot guarantee a specific exposure duration, nor control other aspects of life history. For the purpose of environmental effects monitoring, experimental *in situ* fish caging provides a useful solution to the shortcomings associated with both the laboratory and field studies by creating a more realistic exposure than what is possible in the laboratory while also controlling some of the uncertainties associated with wild fish studies^19,20^. Thus, to reduce the influence of previous contaminant or ecological influences present in the marsh water that might have affected the wild goldfish resident in Cootes Paradise Marsh (CPM), we caged male goldfish purchased from a hatchery and placed them in cages along a gradient of exposure to the Dundas WWTP outfall within CPM (3 sites, CPM1, CPM2, CPM3; Fig. 1). We also compared these caged fish (along the gradient) to fish caged at a reference location in Jordan Harbour, a conservation area in Lake Ontario (JH; Fig. 1). To relate alterations in behaviour to the WWTP exposure, we measured the concentration of select PPCPs in both water and fish plasma. We also measured the concentrations of select monoamine neurotransmitters in fish plasma as part of the larger targeted metabolomic analysis focussed on connecting the exposure to physiological responses that could explain behavioural effects. To our knowledge, this is one of the first studies to attempt to link WWTP effluent exposure directly to the bioaccumulation of PPCPs and subsequent molecular and behavioural alterations.

## Results

### Behavioural assays

**Figure 2:**
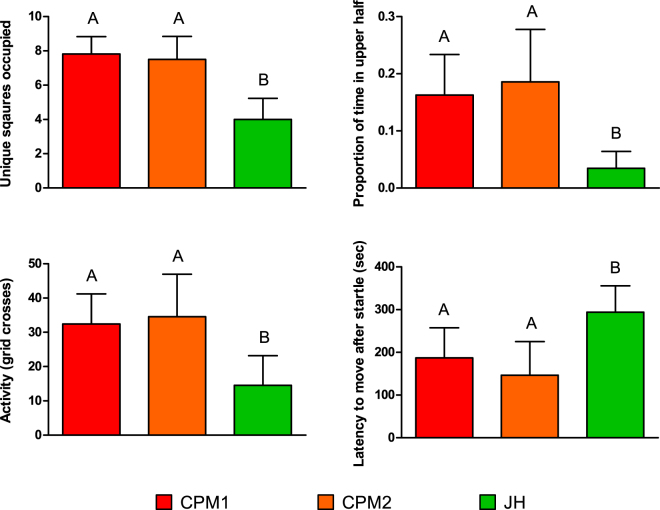
The behaviour displayed in the assays performed on caged goldfish from Cootes Paradise Marsh (CPM) and Jordan Harbour (JH) (n = 48, Bar = Mean response, Error Bars = 95% CI). Differences in unique squares occupied and latency to move after startle were assessed using linear models. The proportion of time in upper half was assessed using a beta regression, and activity (grid crosses) using negative binomial generalized linear models.

Fish exposed to wastewater, at CPM1 and CPM2, were more active and took less time to return to normal after a startle when compared to fish from the reference site (Fig. 2). Fish from these wastewater-impacted sites (CPM1and CPM2) crossed more grid squares (were more active) than fish from the reference site (JH), (Negative Binomial GLM: JH vs CPM1, Z = −2.90, p = 0.0037, JH vs CPM2, Z = −3.13, p = 0.0017; CPM2 vs CPM1, Z = 0.23, p = 0.82). Fish from cages near wastewater effluent were also more exploratory than fish held at the reference site, occupying more unique squares during the activity trial (Linear model: JH vs CPM1, t = −4.77, p < 0.0001; JH vs CPM2, t = −4.38, p < 0.0001; CPM2 vs CPM1, difference = −0.39, p = 0.70) and spent more time in the upper half of the water column, a high-risk area for predation in the wild (Beta Regression: JH vs CPM1, Z = −2.41, p = 0.016; JH vs CPM2, Z = −2.63, p = 0.0087; CPM2 vs CPM1, Z = 0.22, p = 0.83). Fish from all the caging sites responded similarly to the marble drop (startle-response test) by darting (47% of fish), freezing (48%), or remaining active (5%), with site having no effect on the startle response employed (Fisher Test, p = 0.15). However, after being startled, fish from sites near wastewater effluent began to move again and explore faster than did the fish from the reference site (Linear model: JH vs CPM1, t = 2.28, p = 0.028; JH vs CPM2, t = 3.14, p = 0.003; CPM2 vs CPM1, t = −0.86, p = 0.39). Caging site had no effect on feeding rates, nor did it impact the number of feedings attempts (Negative binomial GLM: JH vs CPM1, Z = −0.48, p = 0.63, JH vs CPM2, Z = 0.36, p = 0.71; CPM2 vs CPM1, Z = −0.84, p = 0.40) or feeding successes (Negative binomial GLM: JH vs CPM1, Z = −0.69, p = 0.49, JH vs CPM2, Z = −1.83, p = 0.066; CPM2 vs CPM1, Z = 1.15, p = 0.25). There were no differences between CPM1 or CPM2 for any of the behavioural response tests.

### Neurotransmitters in plasma

**Figure 3:**
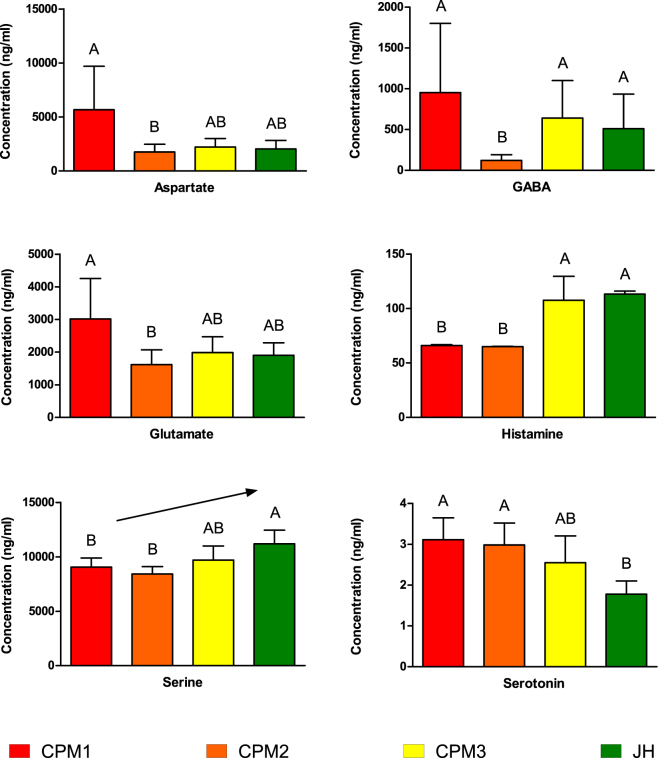
Mean plasma concentrations of neurotransmitters in caged goldfish from Cootes Paradise Marsh (CPM1, CPM2, and CPM3) and the reference site, Jordan Harbour (JH). A and B denote groups with significantly different means (ANOVA, n = 25, p < 0.05, Tukey’s Multiple Comparison, α = 0.05). Error Bars = 95% CI. Arrow indicates a significant linear trend.

Of the 10 neurotransmitters measured in plasma (which were analyzed as part of the metabolomics panel employed on all plasma samples, see companion paper by Simmons *et al*.^18^), the concentrations of 6 were significantly affected by caging location (Fig. 3). Serotonin levels were higher in the plasma of fish caged at sites nearer the effluent outflow (CPM1 and CPM2) compared with fish caged at reference site (JH; ANOVA, F_3,52_ = 4.993, p = 0.0041, Tukey’s multiple comparison test, α = 0.05). Aspartate and glutamate were strongly positively correlated to each other (Pearson r = 0.9204, p < 0.0001) and present in greater concentrations in plasma of fish caged close to the effluent outflow (CPM1) compared to fish caged further downstream at CPM2 and CPM3 (ANOVA, F_3,95 = _3.379 for aspartate and F_3,95 = _3.085 for glutamate, p = 0.02 for aspartate and p = for glutamate, 0.03, Tukey’s multiple comparison test, α = 0.05). Histamine was lower in plasma of goldfish caged at the two sites downstream from wastewater effluent (CPM1 and CPM2) compared with fish at from the marsh proper (CPM3) and the reference site (JH) (ANOVA, F_3,94_ = 22.83, p < 0.0001, Tukey’s multiple comparison test, α = 0.05) as was serine (ANOVA, F_3,95_ = 5.466, p = 0.0016, Tukey’s multiple comparison test, α = 0.05), with an increasing linear trend across the exposure gradient (ANOVA, test for linear trend, p = 0.0011). GABA was significantly lower in plasma of goldfish at the CPM2 site compared to fish caged at other locations (Kruskal-Wallis statistic = 15.32, Dunn’s multiple comparison text, α = 0.05).

### Pharmaceuticals and Personal Care Products in Plasma

**Figure 4:**
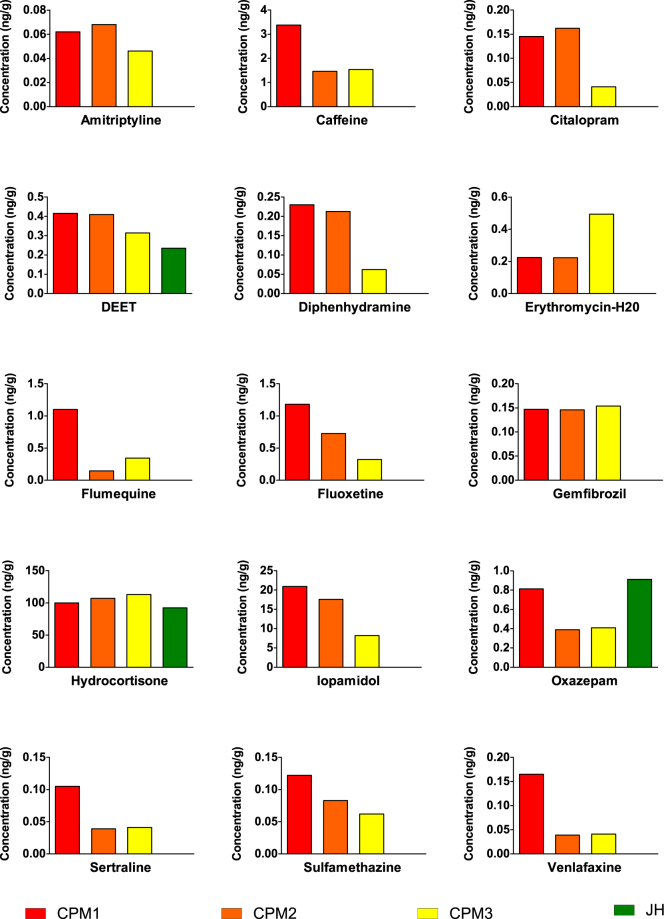
Pharmaceuticals detected in plasma of male caged goldfish after 21 days of deployment in Cootes Paradise Marsh (CPM1, CPM2, and CPM3) and the reference site, Jordan Harbour (JH) (each bar represents a single value from pooled plasma of 25 goldfish). Data presented in this figure are adapted from the companion manuscript by Muir *et al*.^17^.

The PPCP data presented here were extracted from a larger dataset which is covered in depth in the companion manuscript by Muir *et al*.^17^. Out of 127 targets that were analyzed, 15 PPCPs were detected in the plasma of caged goldfish from CPM. Six of these were antidepressant drugs or their metabolites (amitriptyline, citalopram, fluoxetine/norfluoxetine, sertraline, and venlafaxine), 3 were antibiotics or antimicrobials (erythromycin-H_2_O, flumequine, and sulfamethazine). The other PPCPs detected were a stimulant (caffeine), an insect repellent (DEET), a lipid regulating drug (gemfibrozil), a steroid anti-inflammatory (hydrocortisone), a medical contrast agent (iopamidol), and a benzodiazepine (oxazepam) which is often prescribed to relieve anxiety and insomnia (Fig. 4). Three of these PPCPs (DEET, hydrocortisone, and oxazepam) were also detected in fish from the reference site, Jordan Harbour. The concentrations of most of the PPCPs detected in plasma reflected a decreasing trend that corresponded with the exposure gradient of the caging locations within CPM (CPM1 > CPM2 > CPM3 > JH), except for amitriptyline, citalopram, erythromycin-H_2_O, gemfibrozil, and hydrocortisone.

The mean concentration of aspartate in goldfish plasma caged at each location positively correlated with the concentration of flumequine (Pearson r = 0.9659, p = 0.0341) and venlafaxine (Pearson r = 0.9572, p = 0.0428) (Table 1). The mean concentration of histamine in goldfish plasma caged at each location negatively correlated with the concentration of citalopram (Pearson r = −0.9894, p = 0.0106), DEET (Pearson r = −0.9552, p = 0.0448), and diphenhydramine (Pearson r = −0.9872, p = 0.0128) (Table 1). The mean concentration of serine in goldfish plasma caged at each location correlated negatively with the concentration of Σamitriptyline (Pearson r = −0.9851, p = 0.0149) and DEET (Pearson r = −0.9553, p = 0.0447) (Table 1). The mean concentration of serotonin in goldfish plasma caged at each location correlated positively with the concentration of Σamitriptyline (Pearson r = 0.9809, p = 0.0191), DEET (Pearson r = 0.9835, p = 0.0165), iopamidol (Pearson r = 0.9897, p = 0.0193), and sulfamethazine (Pearson r = 0.9735, p = 0.0265) (Table 1).

**Table 1:** Correlation matrix for PPCPs and mean neurotransmitter concentrations detected in goldfish plasma. Pearson r correlation coefficients in bold font were significantly correlated (p-value < 0.05).

	Pearson r correlation coefficient						p-value					
	Aspartate	GABA	Glutamate	Histamine	Serine	Serotonin	Aspartate	GABA	Glutamate	Histamine	Serine	Serotonin
ΣAmitriptyline	0.3465	−0.0359	0.2374	−0.8390	**−0.9851**	**0.9809**	0.6535	0.9641	0.7626	0.1610	**0.0149**	**0.0191**
Caffeine	0.8506	0.5832	0.7970	−0.7159	−0.6817	0.8701	0.1494	0.4168	0.2030	0.2841	0.3183	0.1299
Citalopram	0.4167	−0.1165	0.2721	**−0.9894**	−0.9354	0.9274	0.5833	0.8835	0.7279	**0.0106**	0.0646	0.0726
DEET	0.5003	0.0257	0.3736	**−0.9552**	**−0.9553**	**0.9835**	0.4997	0.9743	0.6264	**0.0448**	**0.0447**	**0.0165**
Diphenhydramine	0.5459	0.0324	0.4118	**−0.9872**	−0.9088	0.9462	0.4541	0.9676	0.5882	**0.0128**	0.0912	0.0538
Erythromycin-H2O	0.0068	0.1584	0.0361	−0.0206	−0.4586	0.4691	0.9932	0.8416	0.9639	0.9794	0.5414	0.5309
Flumequine	**0.9659**	0.7950	0.9476	−0.5418	−0.4168	0.6706	**0.0341**	0.2050	0.0524	0.4582	0.5832	0.3294
ΣFluoxetine	0.7704	0.3404	0.6689	−0.9080	−0.7964	0.9245	0.2296	0.6596	0.3311	0.0920	0.2036	0.0755
Gemfibrozil	0.3044	0.0995	0.2424	−0.6084	−0.8788	0.8988	0.6956	0.9005	0.7576	0.3916	0.1212	0.1012
Hydrocortisone	−0.2168	−0.1760	−0.2278	−0.1385	−0.5993	0.5130	0.7832	0.8240	0.7722	0.8615	0.4007	0.4870
Iopamidol	0.5970	0.1352	0.4780	−0.9490	−0.9181	**0.9807**	0.4030	0.8648	0.5220	0.0510	0.0819	**0.0193**
Oxazepam	0.4621	0.5238	0.5096	0.2035	0.6348	−0.4510	0.5379	0.4762	0.4904	0.7965	0.3652	0.5490
Sertraline	0.8920	0.6261	0.8414	−0.7041	−0.6311	0.8349	0.1080	0.3739	0.1586	0.2959	0.3689	0.1651
Sulfamethazine	0.6926	0.3319	0.6048	−0.8443	−0.8596	**0.9735**	0.3074	0.6681	0.3952	0.1557	0.1404	**0.0265**
Venlafaxine	**0.9572**	0.7012	0.9144	−0.6620	−0.5090	0.7404	**0.0428**	0.2988	0.0856	0.3380	0.4910	0.2596

## Discussion

We observed that exposure to WWTP effluent altered behaviour in male goldfish that spent only three weeks caged in a freshwater environment receiving WWTP effluents. Those behavioural effects were likely mediated in-part by changes observed in neurotransmitter levels in blood plasma of exposed fish. Goldfish exposed to WWTP effluent *in situ* had increased levels of plasma serotonin, aspartate, and glutamate, and were more active, more exploratory, and took less time to resume motion after a startle than goldfish exposed to a reference site lacking these WWTP effluent inputs. These results point to a reduced anxiety (i.e. an anxiolytic effect) making fish behave more boldly.

Behavioural effects have most often been observed in fish during laboratory exposures to single pharmaceuticals at concentrations that are above what has been detected in the natural aquatic environments^21–23^. However, when fish are exposed to WWTP effluents in the wild, they encounter a complex mixture of trace-level contamination. Complicating this situation further, mixture components of PPCPs and their concentrations in WWTP effluents often vary temporally and spatially^24,25^. Little is known about the effects of chronic trace-level pharmaceutical mixtures on fish physiology and behaviour, and the interaction between drugs and contaminants can be complex (additive, synergistic, and/or antagonistic)^26^, therefore it is difficult to attribute cause-and-effect directly to any one substance. In addition to the PPCPs present in WWTP effluents, recipient environments often contain many other contaminants from industrial discharges, legacy contaminants, surface water run-off, agricultural sources and atmospheric transport. Despite these complications, studies examining the effects of WWTP effluent exposures *in situ* are needed to better understand real world effects on aquatic organisms. Also to our knowledge, no study to date has measured bioaccumulation of PPCPs in circulating blood plasma of caged fish as we have done in the present study, although there have been studies that have quantified PPCPs in plasma^27,28^ and tissues^29,30^ of wild fish (see companion paper by Muir *et al*. for more detailed discussion^17^). Thus, while it is normally difficult to directly identify what pollutants present in the complex mixture of a WWTP effluent elicit behavioural changes: our approach in the present study helped us to narrow the field down to a few likely candidates.

Initially we were able to rule-out the influence of oxazepam, and hydrocortisone because both PPCPs bioaccumulated in goldfish at all caging locations including the reference site and were not correlated with the mean concentrations of any of the neurotransmitters – and thus it is unlikely that they would be the causal agents for observed *differences* in neurotransmitter levels and behaviour. We can also eliminate caffeine and gemfibrozil as having behavioural effects, even though they were both detected in the plasma of goldfish caged downstream from the WWTP effluent in CPM and not at the reference site, Jordan Harbour. While caffeine has a stimulating effect in humans, caffeine is known to have an anxiogenic effect in zebrafish (*Danio rerio*), which normally results in more cautious and less exploratory behaviour^31,32^. However, in the present study, we observed anxiolytic effects, and thus we surmised that it was unlikely that caffeine exposure would have caused the behavioural changes in our goldfish. Adding further weight to this assumption, we found no correlation between caffeine and mean neurotransmitter concentrations in plasma. Gemfibrozil is classified as a fibrate drug, which lowers levels of circulating lipids (triglycerides and low density lipids) by activating the peroxisome proliferator-activating receptor alpha (PPARα) which then causes increased synthesis of lipoprotein lipase^33^. Studies have reported that gemfibrozil could reduce androgen synthesis *in vitro* ^34^ and *in vivo* ^35^, and also that it reduces swimming activity^36^. However, we observed increased swimming activity in fish that were part of the present study, and did not observe a correlation between neurotransmitters and gemfibrozil plasma concentrations. Thus we concluded that gemfibrozil exposure was likely not a contributing factor affecting behaviour.

Flumequine was significantly positively correlated with aspartate; sulfamethazine was significantly positively correlated with serotonin, while erythromycin-H_2_O levels did not correlate with the concentration of any neurotransmitter. To our knowledge, no published studies currently exist that describe the effects of antibiotics and antimicrobials on fish behaviour (or on animal behaviour in general). Similarly, iopamidol levels were significantly positively correlated to serotonin concentrations in goldfish plasma, but there is no information regarding the potential behavioural or toxicological effects of iopamidol – although there is indirect evidence that chronic exposure to this molecule may affect the hypothalamic–pituitary–thyroid axis^37^. DEET was also significantly positively correlated to serotonin, and was significantly negatively correlated to histamine and serine. DEET is generally considered toxic to fish only at concentrations thousands of times greater than levels observed in freshwaters^38^, and there have been no controlled behavioural studies of DEET with fish. In the absence of further information, it is difficult to determine if flumequine, sulfamethazine, iopamidol, and/or DEET could be causative agents of the behavioural alterations we observed. We recommend that these knowledge-gaps be addressed in future behavioural studies.

Out of the remaining six PPCPs that were detected in their plasma, male goldfish accumulated five antidepressants (amitriptyline, fluoxetine - including metabolite norfluoxetine, citalopram, sertraline, and venlafaxine) in their plasma when caged closer to the WWTP outfall. The levels of plasma amitriptyline and venlafaxine were significantly positively correlated with mean concentrations of aspartate and serotonin, respectively. Fluoxetine, citalopram, and sertraline are all selective-serotonin reuptake inhibitors (SSRIs), and amitriptyline and venlafaxine are selective-serotonin norepinephrine reuptake inhibitors (SNRIs). These compounds are thought to increase serotonin concentrations by preventing its reuptake from the synaptic cleft by the serotonin transporter (SERT)^39^. As drug targets, SERTs are relatively well conserved across vertebrate species, including fish^40^. This suggests that SSRIs designed for human use would have similar modes of action in teleost fish. Supporting the notion that SSRIs could have caused behavioural changes in our goldfish, increased plasma serotonin levels were observed in fish caged at the WWTP effluent exposed sites, indicating inhibition of serotonin uptake by SERTs likely occurred. Several studies have also observed increases in activity and anxiolytic effects in fish during laboratory exposure to SSRIs supporting our observations – for instance; zebrafish exposed to fluoxetine spent more time at the top of the tank and were more active^41^. In guppies (*Poecilia reticulate*) exposed to fluoxetine, response time to a predator was delayed in males and females^42^, and fluoxetine-exposed fathead minnow (*Pimephales promelas*) were more exploratory in a novel environment^40^. Similarly, fathead minnows exposed to sertraline were more active and less likely to seek shelter in a brightly lit environment^43^. Three-spine stickleback (*Gasterosteus aculeatus*) were more active and exploratory when exposed to citalopram^23^. Endler guppies (*Poecilia wingei*) were more likely to move in a novel environment after citalopram exposure^44^. Venlafaxine has been identified as a neuroendocrine disruptor^45^ that can alter predator avoidance^46^ and predation behaviour^47,48^. At the time of this study, there is no published data available about amitriptyline and fish behaviour. Nonetheless, the observations in the present study reflect the logical premise that SSRIs and SNRIs could affect fish behaviour, and are supported by increased levels of plasma serotonin, and reflect the behavioural results of other published studies.

Goldfish in the present study also accumulated more of the antihistamine diphenhydramine when caged closer to the effluent outfall. Predictably, plasma concentrations of histamine were negatively correlated with accumulated levels of diphenhydramine. This is relevant to behaviour because diphenhydramine has multiple modes of action; as an inverse agonist of the histamine receptor (H1)^49^, an antagonist of muscarinic acetylcholine receptors (mAChR^50^), and as a SERT reuptake inhibitor^51^. In fish, exposure to diphenhydramine in the lab has had largely sedating effects on behaviour. Crucian carp (*Carassius carassius*) exposed to ≥ 21.7 µg/L displayed suppressed feeding rate and reduced movement in an open tank^40,52^. Locomotion was reduced in zebrafish exposed to much higher (>1000x) nominal concentrations of^53^diphenhydramine^43^. Berninger *et al*.^44,54^ also found that diphenhydramine reduced feeding behaviours in fathead minnows at exposure of ≥ 5.6 µg/L, but that this change in behaviour did not affect growth during subchronic exposures. In the present study, exposure levels were an order of magnitude lower than in the laboratory exposures discussed above – which may explain why the sedative effects of diphenhydramine were absent in our goldfish. However, because we observed lowered histamine in goldfish, plasma levels of diphenhydramine must have been high enough to have contributed to inverse agonistic effects at the H1 receptor. Furthermore, the presence of diphenhydramine in a mixture with other SSRIs, as we know is the case with the goldfish caged near the WWTP in the present study, can have an additive effect enhancing the inhibition of serotonin reuptake at various SERT isoforms^55,56^. Thus, it is possible that the levels of diphenhydramine that our goldfish experienced *in situ* had an additive effect on serotonin-linked behaviour.

The findings of the present study add to the growing body of literature demonstrating that WWTP effluent affects fish behaviour. Previously published studies reported reductions in behaviours such as territory defence, nest building, courtship, and swimming performance when fish were exposed to WWTP effluents^10–12,15^. The majority of these studies noted differences in behaviour between male and female fish, and based upon hormone and/or vitellogenin (Vtg) biomarker induction, they concluded that reduced reproductive behaviour(s) in male fish were likely due to the presence of environmental estrogens. However, in the present study we did not observe induction of Vtg in caged male goldfish^18^, and thus we do not suspect that the environmental estrogens which may be present in the wastewater effluents entering CPM were at high enough concentrations to play a role in the behavioural effects we observed. In addition, goldfish appear to be less sensitive to environmental estrogens and estrogenic effluents^13^. Instead, the WWTP effluent exposed goldfish in the present study were more active and exploratory, which is similar to the observations of Saaristo *et al*.^14^, who showed that male mosquitofish collected from the wild near a WWTP outfall more actively courted females than males from a reference location, even though the levels of environmental estrogens and androstenedione were not different at either location. In our study we observed increased concentrations of serotonin in fish caged closer to WWTP effluents, and although we did not assay reproductive behaviour, observe effects on gonadal somatic index^18^, nor find evidence of reproductive endocrine disruption^18^, serotonin is intimately involved in fish reproduction^57^. Serotonin is thought to stimulate the release of gonadotropin-releasing hormone (and gonadotropin), increase gonad maturation, and modulate reproductive and social behaviours in fish^57^. Induction of Vtg by the SSRI fluoxetine was previously observed in goldfish, which was enhanced in mixture with ethinylestradiol^58^, demonstrating the potential for serotonin to interact with reproductive function. Thus, it is possible that the increased serotonin levels we measured in our goldfish could affect their reproductive behaviour and development. Our experimental design used only males and exposed the fish for three weeks during the spawning season: it was not designed to detect changes in reproductive behaviour. In future, it would be useful to expand the current caging strategy to include both females and males so that we could observe if the water at Cootes Paradise influences the behaviour of breeding pairs.

## Conclusions

After three weeks of exposure in Cootes Paradise Marsh, a large wetland that receives WWTP effluent, we observed an accumulation of PPCPs from trace levels in water into fish plasma, at concentrations that were sufficient to alter neurotransmitter levels and behavioural responses. CPM is a unique location where water inputs to the marsh are dominated by WWTP effluents and especially so during the summer when there is little rainfall. Thus, CPM offers an ideal opportunity to study the effects of WWTP effluents. Although CPM may not be representative of all recipient environments, effluent-dominated watersheds are common around the world; they represent a “worst-case” scenario for evaluating the impacts of wastewater effluents^59^. Our caging design, along a gradient of exposure, and including bioanalytical PPCP and monoamine neurotransmitter analyses allowed us to link behavioural endpoints specifically to exposure. WWTP effluents can also contain other contaminants, such as pesticides, which our study did not evaluate, and which could have had effects on molecular and behavioural responses. Our results strongly indicate, however, that the combined effects of multiple PPCPs present in WWTP effluents are likely to affect wild fish populations by reducing their anxiety and resulting in altered swimming activity and response behaviour.

## Methods

### Caged goldfish deployments

This study complied with Canadian Council on Animal Care guidance and it was approved by the GLLFAS/WSTD Animal Care Committee (Government of Canada) and the McMaster Animal Research Ethics Committee. We used only one sex of goldfish to eliminate sex as a confounding factor: we specifically selected male goldfish to test the potential estrogenic influence of the WWTP. We purchased male goldfish from AQUAlity Tropical Fish Wholesale, Inc. (Mississauga, ON) and subsequently housed them in 1500 L recirculation tanks with flow set for 1 L/gram of fish/day in the Aquatic Life Research Facility (ALRF - Environment Canada and Climate Change, Burlington, ON) for 2 weeks. We treated fish with formalin upon arrival in the lab and fed them Northfin Goldfish Formula (Canadian Aquatic Feeds Ltd, Toronto) at 2% of estimated bodyweight per day. We constructed cages from plastic totes (Rubbermaid Hinged Top Tote, 114 L, Polypropylene, Dimensions: 81 × 51.4 × 44.5 cm), with drilled 5/8” diameter holes for water exchange, and added stainless steel hardware and foam floats that allow us to position the cage 12” above the sediment. We deployed 13 fish per cage, and visited the cages weekly to supplement the fish with 20 g of food per cage. The cages containing goldfish were deployed for 21 days from June 25/26, 2014 to July 16/17, 2014. We had five replicate cages at each of our four sites (see Fig. 1 for map of caging locations). Three of these sites were on a represented a gradient along the plume of the Dundas WWTP outfall: CPM1 at Desjardins Canal (530 m from outfall, nearest), CPM2 at West Pond (975 m from outfall, downstream), and CPM3 at McMaster Landing (3850 m from outfall, furthest). Our fourth site, Jordan Harbour (JH), served as a reference site and was located outside of the CPM watershed (50 km across land). At each site, we measured water temperature, pH, dissolved oxygen, and conductivity (YSI multi-parameter sonde, 6600 series) in duplicate on days 1, 14, and 21 of the exposure period (See Table S1).

### Goldfish plasma collection

We collected plasma as previously described by Simmons *et al*.^18^.

### Plasma pharmaceutical and personal care product analysis

We followed previously described methods described by Muir *et al*.^17^ for detection of PPCPs in goldfish plasma.

### Neurotransmitters and related metabolites

We measured (DL-aspartate, DL-DOPA, dopamine, gamma-Aminobutyric acid (GABA), DL-glutamate, glycine, histamine, phenylethylamine, serine, and serotonin in goldfish plasma using LC-MS/MS (SGS AXYS, Axyomics, Sidney BC). We used internal standards as described in a previously published method^60^ with some alterations which are described in detail in Simmons *et al*.^18^.

### Behavioural assays

We transported goldfish live from the deployed cages at three of the field sites (N = 16 fish per caging site) to McMaster University to undergo behavioural assays (fish from CPM3 were not tested due to time-restriction on behavioural data collection). The remaining fish were used for PPCP, metabolome, and proteome analyses. In the laboratory, we housed fish by caging site in 150 L tanks (H44 cm × W90 cm × D38cm) equipped with natural gravel substrate and a static renewal filter (Aquaclear). We conducted behavioural analyses ~18 hours after transport to allow for recovery from handling and acclimation to the laboratory^51,61^. We conducted three behavioural assays: 1) an activity assay, 2) a startle response assay (that simulated a predation event), and 3) a feeding assay. We recorded all behaviour from behind a 1.8 m vertical opaque barrier that occluded observer movement and limited disturbances during behavioural trial scoring. We conducted all three assays in a 45 L (H33 cm × W51 cm × D28 cm) testing tank, equipped with natural gravel substrate and a static renewal filter (Figure S1). We overlaid a 2-x-3 grid on the front of the aquarium and a 2-x-2 grid on each end of the aquarium to track activity of the focal fish in three dimensions, described in detail below. Additionally, we fixed a 75 cm tube (with a 3 cm diameter) that ended above the top center of the testing tank. The tube allowed us to startle fish using a marble or introduce food in a consistent manner without experimenter disturbance. We began trials when a caged fish was transferred from the housing tank to an experimental tank and then each fish was given 20 minutes to recover from handling, transport and to acclimate to the testing tank.

We sequentially conducted the three behavioural assays. Each assay was 5-minutes in length with 15-minutes habituation in between each assay. During the activity assay, we recorded each time the focal fish entered a new grid square; this occurred when over half the fish’s body and head was in a new square. From these scores, we assessed exploration as the number of unique squares occupied by the fish and the proportion of squares entered that were in the top vs the bottom half of the water column. For the startle response assay, we released a marble (1.5 cm diameter) down the tube to land in the center of the tank. This creates a splash which simulated an aerial predation event, and we measured the fish’s reaction to this disturbance^62^. We recorded the behavioural startle response of each fish (e.g. remained active, freeze, or dart), and the number of seconds it took the fish to resume motion and cross into a new grid square. In our final assay, we measured feeding rates by releasing down the tube 10 sinking fish food pellets (Northfin) into the aquarium. Fish were familiar with this food, as they were fed them in the laboratory prior to caging and during the caging period. We scored the number of feeding attempts (lunges and nips at food) and the number of feeding successes (capture and consumption).

After behavioural testing trial, we removed fish from the testing tank and the tank was given a 50% water change and any remaining food pellets were removed. We then euthanized the fish with an overdose of benzocaine (0.015% solution, Sigma Aldrich) and froze them at −20 °C until later dissection. After later thawing, we measured standard length (snout to caudal peduncle) with callipers accurate to 0.01 cm. We then measured whole body and gonad mass on a digital balance accurate to 0.001 g (Ohaus Adventurer Pro). Fish used for behavioural assays did not differ in body mass (ANOVA: F_2,43_ = 0.24, p = 0.78) or standard length (ANOVA: F_2,43_ = 0.42, p = 0.66) between caging exposure sites.

### Statistical Analyses

For the behaviour assays, we conducted statistical analyses using R (version: 3.2.3, R Core Team 2015). In all analyses, caging site was included as a fixed factor. We analyzed the number of grid crosses (our activity measure), the number of feeding attempts, and the number of successful feeding attempts using negative binomial generalized linear models appropriate for over-dispersed count data. We analyzed the proportion of time fish spent in the upper half of the water column during our activity assay using a beta regression^63^. We analyzed the number of unique squares occupied during the activity assay and the latency for fish to begin moving after being startled using linear models. We analyzed the categorical behavioural response of fish to being startled (i.e. dart, freeze) using a Fisher’s Exact Test. In all tests, no fish were excluded, giving each analysis a sample size of N = 48. For neurotransmitters and related metabolites, we assessed significance of fold change values using Analysis of Variance (ANOVAs) with Fisher’s LSD post-hoc test in Metaboanalyst 3.0 with default settings. To compare groups, we conducted ANOVAs followed by Tukey’s Multiple Comparison test, but when the data did not conform to the assumptions of ANOVA, we performed the Kruskal-Wallis test followed by Dunn’s Multiple Comparison Test. Figures were constructed using Graphpad Prism 5.01.

### Data availability

All data generated or analysed during this study are included in this published article and in Supplementary Information

## Electronic supplementary material

Supplementary Information
