# Supplementary material for: Reduced anxiety is associated with the accumulation of six serotonin reuptake inhibitors in wastewater treatment effluent exposed goldfish Carassius auratus
Source: Sci Rep. 2017 Dec 5;7:17001. doi: 10.1038/s41598-017-15989-z (PMC5717243; doi:10.1038/s41598-017-15989-z)
Supplement: Supplementary file 1 — Supplementary Information [file 41598_2017_15989_MOESM1_ESM.pdf]

# Supplementary Information

Reduced anxiety is associated with the accumulation of six serotonin reuptake inhibitors in wastewater treatment effluent exposed goldfish *Carassius auratus.*

Simmons D[1], McCallum E[2], Balshine S[2], Chandramouli B[3], Cosgrove J[3], Sherry J[1]*.

[1] Aquatic Contaminants Research Division, Water Science and Technology Directorate, Environment Canada, Burlington, ON

[2] Department of Psychology, Neuroscience & Behaviour, McMaster University, Hamilton, ON

[3] Metabolomics Services, SGS AXYS, Sidney, BC

*corresponding author

Table S1: Water quality parameters, presented as mean (range) from duplicate readings at two sampling periods.

| | Exposure site | | | |
|---|---|---|---|---|
| | CPM1 | CPM2 | CPM3 | JH |
| Temperature | 20.45<br>(20.35 – 20.74) | 22.82<br>(22.37 – 22.82) | 20.93<br>(20.45 – 21.52) | 20.94<br>(20.31 – 21.58) |
| pH | 7.86<br>(7.37 – 8.00) | 8.85<br>(8.49 – 9.21) | 7.54<br>(7.22 – 7.72) | 7.66<br>(6.93 – 8.05) |
| DO (mg/L) | 10.66<br>(7.09 - 14.48) | 17.89<br>(15.76 – 20.24) | 6.15<br>(5.31 – 6.71) | 8.10<br>(7.63 – 8.63) |
| Conductivity | 1014.75<br>(964 – 1066) | 1030.25<br>(1002 – 1058) | 745.25<br>(679 – 810) | 458.50<br>(440 – 482) |

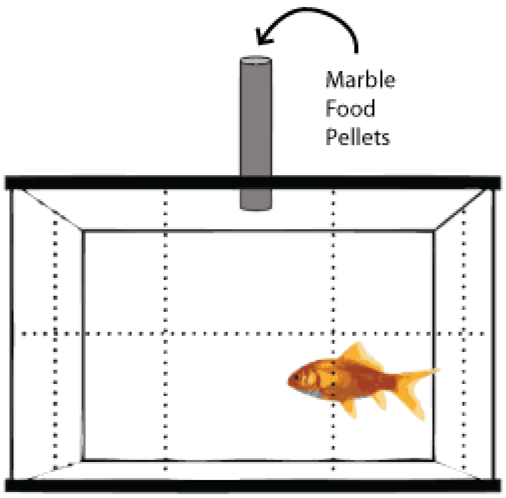


Figure S1. Behavioural assay tank.
